# Tacrolimus in the prevention of adverse pregnancy outcomes and diabetes-associated embryopathies in obese and diabetic mice

**DOI:** 10.1186/s12967-017-1137-4

**Published:** 2017-02-13

**Authors:** Ahmad J. H. Albaghdadi, Melanie A. Hewitt, Samantha M. Putos, Michael Wells, Terence R. S. Ozolinš, Frederick W. K. Kan

**Affiliations:** 1 Department of Biomedical and Molecular Sciences, Faculty of Health Sciences, Queen’s University, Kingston, ON K7L3N6 Canada; 2 PARTEQ Innovations, Queen’s University, Kingston, ON K7L 0E9 Canada

**Keywords:** Diabetes, Immunosuppression, Tacrolimus, Metformin, Embryopathies, Adverse pregnancy outcomes (APO)

## Abstract

**Background:**

T2DM is a high-risk pregnancy with adverse fetal and maternal outcomes including repeated miscarriages and fetal malformations. Despite the established association between placental insufficiency and poor maternal Th1-adaptability to the development of pregnancy complications in T2DM, there have been no established data to assess benefits of pre-pregnancy immunosuppression relative to gestational outcomes in T2DM. We hypothesized that pre-pregnancy macrolide immune suppression can re-establish normal placental development and uterine vascular adaptation in a mouse model of obesity-associated T2DM.

**Methods:**

Fetal live birth rate, postnatal variability, mid-gestational uterine and umbilical flow dynamics and certain morphological features of spiral artery modification were examined in the New Zealand Obese (NONcNZO10/Ltj) female mice (n = 56) weaned to ages of 32 weeks on a 60% calories/g high-fat diet (also referred to as HFD-dNONcNZO), and which received either tacrolimus (0.1 mg/kg s.c. q2d) , its vehicle (castor oil and ethanol) or metformin (in drinking water 200 mg/dL p.o. ad libitum). HFD-BALBc-Rag2/IL2-gc female mice (n = 24) were used as HFD-immunodeficient controls.

**Results:**

Treatment of the HFD-dNONcNZO female mice with tacrolimus improved live birth rates and postnatal viability scores (p < 0.01), normalized OGTT (p < 0.001), inhibited fetal malformation rates, restored morphology of spiral arterial modification; and improved uterine arterial and umbilical blood flow (p < 0.01). Placental production of TNFαand IL16 in the tacrolimus-treated HFD-dNONcNZO dams were restored to non-diabetic levels and the treatment resulted in the inhibition of aberrant monocyte/macrophage activation during pregnancy in the HFD-dNONcNZO dams.

**Conclusions:**

Our present data suggest a casual association between chronic maternal overnutrition and aberrancy in the maternal Th1-immune maladaptation to pregnancy and defective spiral artery modification, placental insufficiency and adverse fetal outcomes in the T2DM subjects. Further safety studies into the use of tacrolimus in the pre-pregnancy glycemic control may be beneficial.

**Electronic supplementary material:**

The online version of this article (doi:10.1186/s12967-017-1137-4) contains supplementary material, which is available to authorized users.

## Background

Although pregnancy complicated by diabetes may result in a normal delivery with few adverse maternal effects or the child’s long-term health, obesity and poor glycemic control during pregnancy can have deleterious maternal and embryo-fetal effects as well as delivery complications [1]. Lifestyle choices, in particular caloric intake, contribute significantly to obesity and increase expression of pro-inflammatory markers that precede the onset of insulin resistance and hyperglycemia associated with T2DM [2]. This apparent immune dysregulation in T2DM suggests that it may be a relevant therapeutic target [2], especially under physiological conditions such as gestation where adverse pregnancy outcomes (APOs) may occur in face of improper glycemic control.

Maintaining adequate glycemic control is the focus of current therapeutic interventions in pregnancies complicated by gestational diabetes mellitus (GDM). However, lowering the risk of APOs has always been a challenge with the use of current therapeutic modalities such as insulin and/or metformin [3, 4]. Although insulin treatment of GDM reduces serious perinatal morbidity and partially improves the woman’s health-related quality of life [5], nonetheless, offspring of obese and diabetic women with adequate symptomatic control of their hyperglycemia have a five to tenfold increased risk of congenital anomalies and a fivefold greater risk of perinatal mortality than non-obese women based upon the population studied [3, 4, 6].

When challenged with a high-fat diet, the New Zealand Obese (NONcNZO10/LtJ) mice become hyperglycemic, hyperinsulinemic and insulin resistant, making them an important tool for investigating the links between metabolic dysregulation and reproductive and developmental defects in the obese and diabetic subjects [7, 8] and reviewed in [9].

While the use of immunosuppressants is primarily indicated to prevent allograft rejection, mounting evidence suggests they may be used safely during pregnancy after solid organ transplant [10, 11]. A recent recommendation suggests that in utero exposure to the immunosuppressant tacrolimus does not increase the risk of major congenital malformations, although there was an increased risk of low birth weight and pre-term birth [12]. Based upon this safety profile and the clinical importance of tacrolimus, it was decided to use this agent to test the hypothesis that immunosuppression can improve pregnancy outcomes in the chronically overfed obese and diabetic murine model of the human obesity-associated T2DM.

## Methods

### Animals

All animal procedures were in accordance with the guidelines of Queen’s University Animal Care Committee. A total of fifty-six recombinant congenic NONcNZO10/LtJ mice (also known as RCS-10 Strain, stock # 004456M) were purchased from the Jackson Laboratory (Bar Harbour, MA). Twenty-four alymphoid mice lacking T, B and NK cells with the genotype BALB/c-Rag2^−/−^Il2rg^−/−^ (also referred to as Rag2^−/−^gc^−/−^) served as control cohorts in the high-fat diet (HFD) experiments, and twenty allowed ad libitum access to a 20% fortified protein pellet diet (5K52 LabDiet^®^ JL, St. Louis, MO) and were referred to as Normal-Fat Diet “NFD” fed mice. All alymphoid mice were bred in house and provided through the Queen’s University Animal Care Services. Mice were housed in decontaminated barrier facility and caged in pairs in ventilated mouse cage racks. To avoid metabolomic inconsistencies [13], animal bedding consisted of recycled heat-treated hardwood Beta Chip^**®**^ and cardboard paper bedding (NEPCO, Northeastern Product Corp., NY).

A 60% kCal high-fat diet (HFD) (D12492, Research Diets Inc., New Brunswick, NJ) was used to feed the NONcNZO10/LtJ mice and their respective control. For the specific purpose of this study and due to variability in disease penetrance, slower disease progression and high unpredictability of diabetes development in male NONcNZO10/LtJ mice [7], only female mice had longitudinal assessment of growth trajectory, glycemic control and reproductive outcomes. Details of the mouse models, therapeutic interventions, dietary and husbandry conditions can be found in Additional file 1: Table S1. The temporal sequence of experimental procedures including administration of high-fat diet, tacrolimus, and metformin are summarized in Additional file 1: Figure S1.

### Preparation of pregnant mice

Allogeneic mouse pregnancies were obtained by mating females with males of the same mouse strain during week 21 HFD which corresponds to week 26 of the mouse age using standard protocol [14]. Briefly, virgin female mice were placed in shoeboxes containing male bedding 48 h prior to cohabitation with males of the same strain, and vaginal plugs detected in the morning (07:00–08:00 a.m.) established GD 0.5 of pregnancy. Due to the current high rate of post-implantation loss in the HFD-dNONcNZO mice, pregnancies in GD 0.5 sperm-positive mice were verified using high-resolution ultrasound (Vevo 770; Visualsonics Inc., Toronto, ON) as described elsewhere in this study.

### Blood glucose monitoring

#### Baseline monitoring

An Ultra glucometer (Accu-Chek^**®**^ Aviva/Roche Diagnostic, Montreal, QC) was used to monitor blood glucose of all mice through tail venipuncture once a week beginning at 7 weeks of age until the time of euthanization. Single drops (~30 μL) of freshly obtained tail vein blood were sufficient to accurately measure blood glucose in all mice. Mice having non-fasting blood glucose concentrations ≥14.49 mmol/L and/or impaired oral glucose tolerance test (OGTT) were considered diabetic and were annotated as HFD-dNONcNZO mice [7]. All HFD-NONcNZO mice with non-fasting blood glucose of ≤6.4 mmol/L were referred to as normoglycemic NONcNZO mice and were excluded from this study.

#### Glucose tolerance test

To conduct the OGTT mice were first fasted for 6 h [15]. A 20% d-glucose (2 mg/g body weight) sterile syrup was administered by oral gavage using a 1 mL D29 gauge insulin syringe (Fisher Scientific Ltd., Montreal, QC) and blood glucose assessed at the following post-glucose challenge time points: 0, 15, 30, 45, 60, 90 and 120 min.

### Test article and dose formulation

Due to the low bioavailability of tacrolimus (14–32%) [16] and data obtained in several pilot attempts at determining the optimum dosage of tacrolimus for use in this particular study, a modified tacrolimus-based monotherapy protocol was developed for use in the HFD-dNONcNZO mice. Tacrolimus (FK506) (Tacrolimus-Astellas Pharma US, Inc., Deerfield, IL) was dissolved in castor oil and ethanol (vehicle for FK506 and untreated groups) and administered to the diabetic HFD-dNONcNZO mice on an alternate day regimen from week 16–19 and week 25–29, respectively (Additional file 1: Figure S1, Table S1). For proper comparisons and adequacy in providing a standard diabetic drug-treated T2DM diabetic mouse cohort for this study, metformin (Metformin Hydrochloride (C4H12CIN5), Catalogue # M258815, Toronto Research Chemicals, Toronto, ON) was selected for use in HFD-dNONcNZO mice to generate genetically matched treated control. Due to the significant first pass effect, metformin doses in diabetic rodents are typically ≥50 mg/day [17]. Using this as a starting point and knowing that average water consumption in the diabetic mouse is ~10–15× the upper limit of normoglycemic mouse water consumption of 7.7 + 0.3 mL/30 g bwt [18], metformin was prepared fresh daily and made available ad libitum (200 mg/dL) per day.

### Ultrasonography and determination of uterine arterial and umbilical blood flow

Transcutaneous Doppler ultrasonography was used to collect and analyze alterations to uterine arterial hemodynamics and umbilical artery waveforms in isoflurane-anesthetized virgin sperm-induced pregnancy (GD 0.5–GD 18.5) among HFD-dNONcNZO and/or those receiving tacrolimus or metformin (n = 3 dams/group/time point). A microultrasound biomicroscope (Vivo770, Visual Sonic Inc., Toronto, Ontario, Canada) and a 30–40 mHz Real-Time Micro Visualization scanhead transducer operating at two frames per second (RMV704 or 707B, VisualSonics Inc, Toronto, ON) were used in recording uterine arterial and umbilical artery blood flow using a previously described method for comparing patterns of uterine and umbilical artery blood flow hemodynamics [19]. Briefly, delivered by a well-fitted facemask, induction of anesthesia was initiated with 5% inhaled isoflurane in oxygen, and this was reduced to 1.5–2% to maintain unconsciousness. On average, the duration of sonography was 5–7 min. The sonographer was blinded to the treatment groups. With insonation angle of <45°, the Brightness mode (B-mode) Doppler and the high frame rate 707B 30 mHz scanhead were used to obtain pulse-wave Doppler (PWD) and record uterine arterial velocity waveforms for offline analysis. Recorded data included: velocities (cm/s; Peak systolic velocity-PSV, end diastolic velocity- EDV and time-averaged mean blood flow velocity-MV), and vascular indices (Pulsatility index (PI) and Resistivity index (RI) according to the equations PI = [PSV − EDV]/MV and RI = [PSV − EDV]/PSV, respectively. Since it has been observed that umbilical artery blood flow velocities are higher at the fetal than the placental end of the cord [20], and for the purpose of consistency in reporting, we strictly measured umbilical blood flow velocities from the placental end of the cord in all experimental groups. The software of the ultrasound machine automatically calculated arterial hemodynamic data after manual or automatic delineation of the Doppler waveforms. Mean values of the hemodynamic data were established by averaging a minimum of five Doppler waveforms at each time point. Finally, the following parameters were implemented in the B-mode recording: the lowest high-pass filter level used was 100 Hz, 2000 ms display window, Doppler gain of 5.00 dB, the pulsed repetition frequency was between 4 and 48 kHz and was set to detect low and high blood flow velocities, respectively.

### Fetal analysis and tissue preparation

Anesthetized (ketamine–xylazine) pregnant mice were euthanized on the mornings of GD18 by cardiac puncture with the heart cut between 09:00 and 11:00 a.m. After performing Cesarean section, the litter size was counted in combination with their location along the length of the respective uterine horn. Both uterine horns were examined for the presence of early and late resorptions. Viable fetuses were identified by virtue of their ability to move and breathe, weighed and examined for gender and fresh gross anomalies [21]. After removal of all membranes from the nose and mouth, the viable fetuses were placed in a 37.5 °C incubator for 1 h, after which the viability of each pup was reassessed. Thereafter, all fetuses that were in the incubator were euthanized and examined for visceral anomalies [21]. Placentas from viable fetuses were weighed and immediately fixed in an alcoholic paraformaldehyde solution (2% wt:vol in 70% ethanol). Skeletons were stained using Alcian Blue and Alizerin Red double staining technique [22]. The International Federation of Teratology Societies (IFTS) glossary [23] nomenclature for description of developmental anomalies was adapted in this report. Hematoxylin and Eosin stained, paraffin-wax embedded placental sections were prepared according to standard protocol [24]. Histological examination of placental sections and calculation of spiral artery wall/lumen ratio were performed by two histologists blinded to the treatment methods.

### Statistical analysis

Data were analyzed with Sigmaplot (Systat Software, Inc., San Jose, CA) and Graph-Pad Prism 6 software. Normal distributions were validated using the Kolmogorov–Smirnov method. Parameters of normally distributed data were expressed, unless otherwise indicated, as mean ± SEM or as mean ± SD using appropriate non-parametric procedures (Fisher Exact test; Kruskal–Wallis and/or one-way ANOVA with interaction effects) followed by Dunn’s multiple comparison test or Mann–Whitney U test or Miller’s procedure for pairwise comparisons of independent parameters. Two-way ANOVA comparing treatment groups with their untreated diabetic or normoglycemic control was also used to determine *p* values for the source of variation across treatments. Linear (parametric) data were assessed by Pearson correlation, whereas nonlinear (non-parametric) data were assessed by Spearman correlation. Glucose tolerance graphs were generated by plotting the recorded glucose data per mouse per minute collection time. One way analysis of variance (ANOVA) followed by Student *t* test was performed to determine alpha values for statistical differences among mean blood glucose values across experimental mouse groups. Due to lack of linearity in the declining glucose concentrations over time during the OGTT, the area under the curve (AUC) measured in the log-linear interpolation method [25] made use of the log average of Δ time interval according to the equation Area _log_ = (C1 − C2)/(*log* C1 − *log* C2)*Δ time. A probability value of *p* ≤ 0.05 was considered statistically significant and *p* values between 0.06 and 0.1 were considered as statistical trend.

## Results

### Maternal parameters

#### Tacrolimus and glucose tolerance in the HFD-dNONcNZO mice

**Fig. 1:**
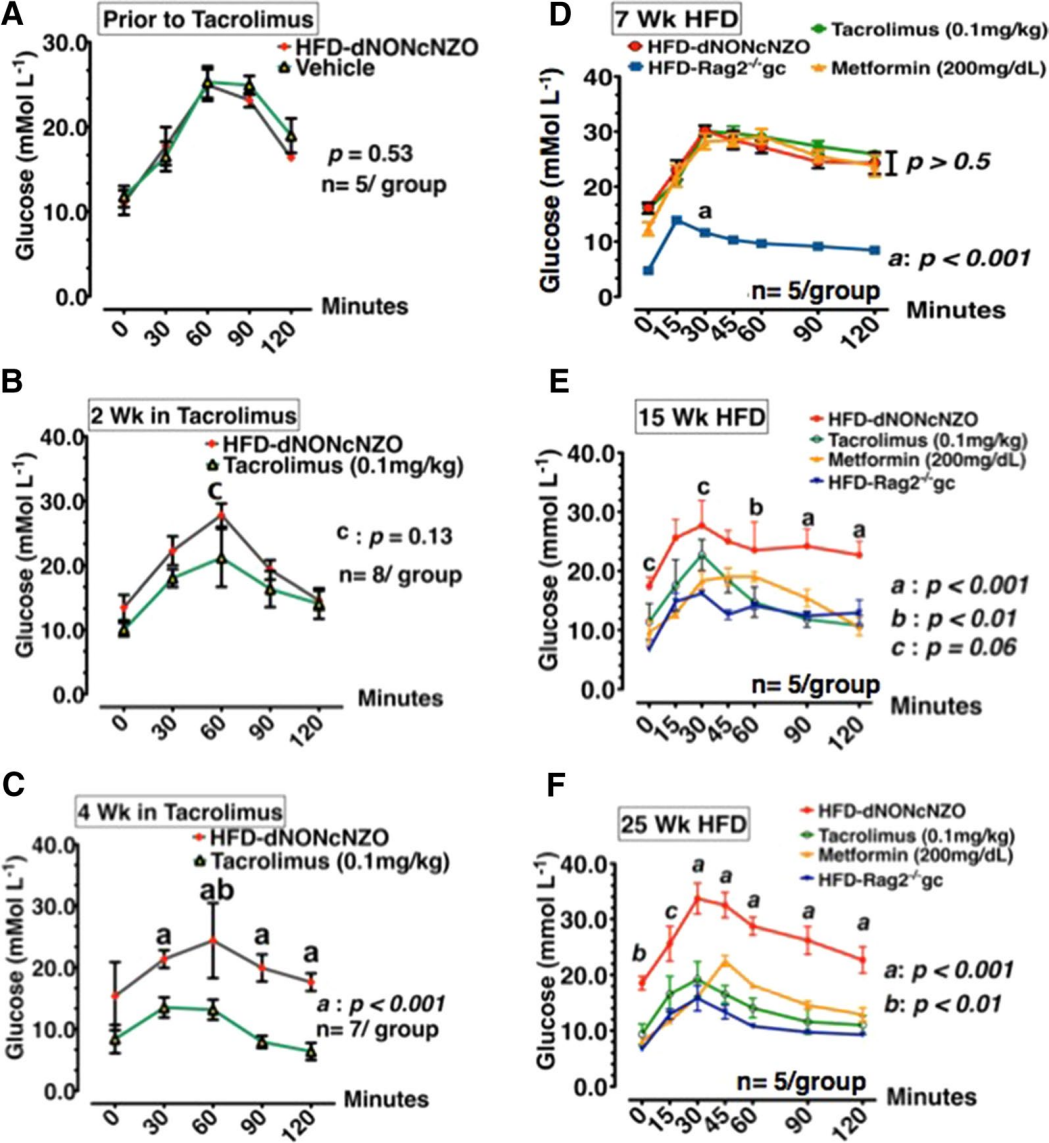
Tacrolimus restored glucose tolerance in the chronically overfed HFD-dNONcNZO dams. **A**–**C** Oral glucose tolerance test (OGTT) performed prior to treatment with tacrolimus (**A**) and after 2 weeks (**B**) or 4 weeks (**c**) of tacrolimus (0.1 mg/kg). **D**–**F** Effect of HFD chronicity and glycemic response to metformin or tacrolimus in the HFD-dNONcNZO mice chronically fed with 60% kcal % HFD for 7 weeks (**D**), 15 weeks (**E**) or 25 consecutive weeks (**F**), respectively. Representative OGTTs were from two sets of experiments (n = 5–8/group) analyzed with one-way ANOVA followed by Dunn’s multiple post hoc test comparing treated mice to HFD-dNONcNZO

After 7 weeks of the HFD, the HFD-dNONcNZO mice developed impaired glucose tolerance which required 4 consecutive weeks of therapeutic intervention with tacrolimus (0.1 mg/kg) to restore proper glycemic response at OGTT as shown in Fig. 1A–C. Therefore, tacrolimus was administered in two consecutive therapeutic interventions, each of 4 weeks in duration for the period spanning HFD weeks 15–18 and HFD weeks 25–28, respectively. This significantly improved glucose tolerance as shown in Fig. 1D–F, and lowered the AUC glucose in OGTT in a time-dependant manner (Additional file 1: Figure S2A–C). Treated groups only had moderate hyperglycemia detected during mid-gestation at GD10.5 (9.36 ± 0.88 tacrolimus vs 8.08 ± 0.76 mmol/L metformin) as opposed to the significant hyperglycemia observed in untreated diabetic HFD-dNONcNZO dams (18.53 ± 0.58 mmol/L) (p < 0.001). However, except for some subtle differences between the metformin- and the tacrolimus-treated and their corresponding untreated HFD-dNONcNZO dams, lower blood glucose values were detected at necropsy at GD 18.5 among the tacrolimus-treated mouse cohorts (Table 1).

**Table 1:** Maternal and reproductive parameters at the time of cesarean section in the HFD-dNONcNZO mice and those receiving tacrolimus or metformin

	Maternal BWt (g)	Mean BG (mmol/L)	No. of litters	Litters with viable fetuses	No. total implants	No. total fetuses	Total viable fetuses	% viable fetuses	Implantation sites/litter (mean ± SD)	No. resorption/litter (mean ± SD) % (mean ± SD)	No. fetuses/litter (mean ± SD)	Viable fetuses/litter at necropsy (mean ± SD) (%) (mean ± SD)	Fetal viability 1 h post necropsy (%) viable at parturition	Male:female ratio	Fetal wt. (m)	Placental wt. (g)
HFD-dNONCNZO	57.5 ± 8.1	14.67 ± 1.96	4	3	21	10	3	30	5.25 ± 1.17	2.75 ± 0.50 (55 ± 10.6)	2.5 ± 1.29	0.75 ± 0.50 (33.0 ± 19.1)	0	50:50	1.30 ± 0.57	0.213 ± 0.14
TACRO (0.05 mg/kg)	38.7 ± 0.81^a^	7.60 ± 0.71^a^	3	3	26	23	22	96	8.67 ± 0.58^a^	1.0 ± 1.73 (16.5 ± 19.1)	7.67 ± 1.53^a^	7.33 ± 1.15 (96.3 ± 7.78)	91.0 ± 10.1^b^	40:60	1.01 ± 0.325	0.181 ± 0.050^a^
TACRO (0.1 mg/kg)	45.07 ± 3.72	7.13 ± 1.59^a^	3	3	23	19	18	95	7.13 ± 1.53	1.33 ± 0.71 (16.7 ± 6.5)	6.33 ± 1.53^a^	6.0 ± 2.0 (93.3 ± 11.5)^b^	89.0 ± 19.1^b^	62:38	1.37 ± 0.209	0.204 ± 0.024
Metformin (200 mg/dL)	54.22 ± 1.63^a^	9.62 ± 2.63^a^	3	3	27	21	17	81	9.0 ± 2.05^a^	2.0 ± 2.65 (18.7 ± 23.5)	7.00 ± 1.00^a^	5.67 ± 1.15 (82.0 ± 40.1)	80.0 ± 41.5^b^	52:48	0.847 ± 0.33^b^	0.1684 ± 0.029^b^

HFD-dNONcNZO data are presented as mean ± SD

*TACRO* tacrolimus, *BG* blood glucose

^a^Significantly different from HFD-dNONcNZO, p < 0.05 Kruskal–Wallis

^b^Significantly different from HFD-dNONcNZO, p < 0.01 Kruskal–Wallis

#### Tacrolimus protects against fetal demise and reproductive health adversities in the HFD-dNONcNZO dams

**Fig. 2:**
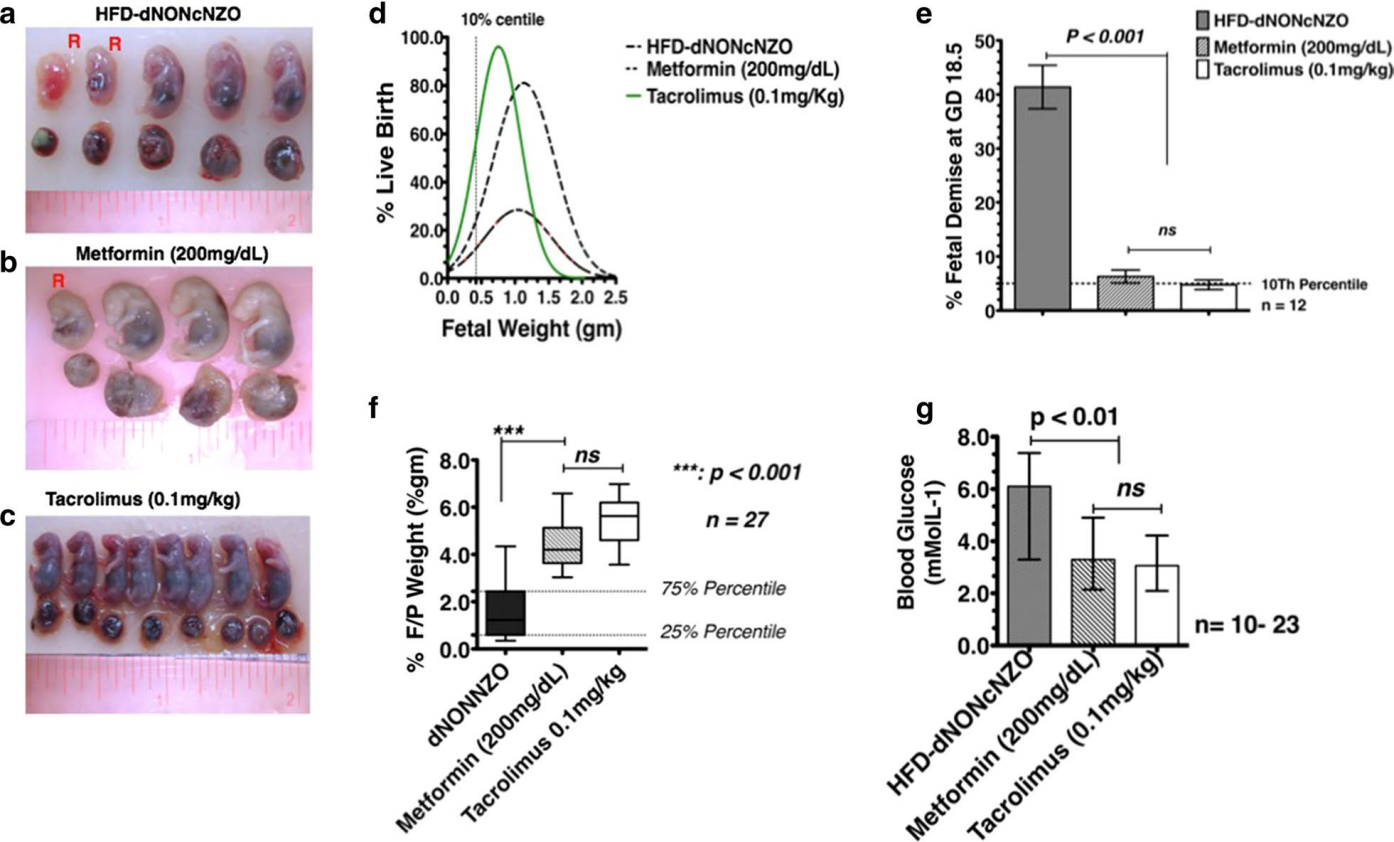
Low fetal viability and high rate of fetal demise were characteristics of pregnancy in the HFD-dNONcNZO dams. **a**–**c** Findings at necropsy at gd18.5 for the common gross manifestations of GDM among the HFD-dNONcNZO dams typically expressing low fetal viability and higher rates of fetal resorption. Notwithstanding its effect on birth weight, treatment with tacrolimus enhanced live birth rate (**d**), lowered percentage of demise (**e**) and enhanced fetal/placental (F/P) weight ratio (**f**). Reference values for random blood glucose were also detected among pups delivered to tacrolimus-treated dams (**g**). n = a minimum of 7 dams/group with variable numbers of fetuses/pups examined. *p* < 0.001 in **d**

The higher maternal weights (Table 1), elevated non-fasting glucose levels and impaired OGTT in the untreated HFD-dNONcNZO dams translated to poorer pregnancy and progeny outcomes compared to the treated HFD-dNONcNZO groups receiving either tacrolimus or metformin. Reduced implants was a characteristic feature of APOs with fewer implants per litter in untreated HFD-dNONcNZO (5.25 ± 1.71 implants/dam) as opposed to the tacrolimus-treated dams (8.67 ± 0.58 and 7.13 ± 1.53 implants/dam) or those receiving metformin (9.0 ± 2.05 implants/dam), (Table 1). In addition, the numbers of viable fetuses per litter and individual fetal birth weight were also significantly reduced in untreated dams (Table 1: compare 2.5 ± 1.29 vs 7.67 ± 1.53, 6.33 ± 1.53 or 7.00 ± 1.00) and Fig. 2a–d. Viable fetuses at the time of necropsy, expressed as a percent total fetuses per litter was also significantly reduced in untreated dams (33.0 ± 19.1) when compared to those treated with tacrolimus (0.05 or 0.1 mg/kg) or metformin (Table 1: 96.3 ± 7.78 and 93.3 ± 11.5 in the tacrolimus-treated and 82.0 ± 40.1 in the metformin-treated dams). Indeed, whereas between 80 and 89% of the fetuses were still viable 1 h post necropsy in the treated groups (Fig. 2d), fetal viability among HFD-dNONcNZO dams was less than 20% at necropsy at GD 18.5 (Fig. 2d), and none of the HFD-dNONcNZO progeny was viable 1 h post-necropsy. The resorption rate, when calculated as a percent total implants per litter and percentage of fetal demise at GD 18.5 among untreated dams were significantly elevated in the HFD-dNONcNZO mice (Table 1: compare 55.0 ± 10.6 vs 16.5 ± 19.1, 16.7 ± 6.5 or 18.7 ± 23.5) (see also Fig. 2e). The Placentas from HFD-dNONcNZO dams were heaviest, whereas the lowest placental weights came from low-dose tacrolimus- and metformin-treated dams. Although the higher fetal/placental (F/P) weight ratio in the treated groups (Fig. 2f) was indicative of better in utero fetal health, nonetheless, fetal body weights followed a similar trend, but unlike the placental findings these trends were not statistically significant (p ≥ 0.07). Finally, contrasting the human studies in which the rate of neonatal hypoglycemia among women with elevated BMI were 3% [26], fetal blood glucose at necropsy in the present study was found to be significantly elevated in the untreated HFD-dNONcNZO dams (6.69 ± 0.41 mmol/L) (Fig. 2g). Normal values were detected in the treated groups irrespective of the treatment modality (3.15 ± 0.27 mmol/L in the tacrolimus-treated vs 3.56 ± 0.36 mmol/L in the metformin-treated dams) (Fig. 2g).

#### Tacrolimus-mediated prevention of fetal demise is mechanistically linked to restored uterine artery physiology and umbilical flow during gestation in treated HFD-dNONcNZO mice

**Fig. 3:**
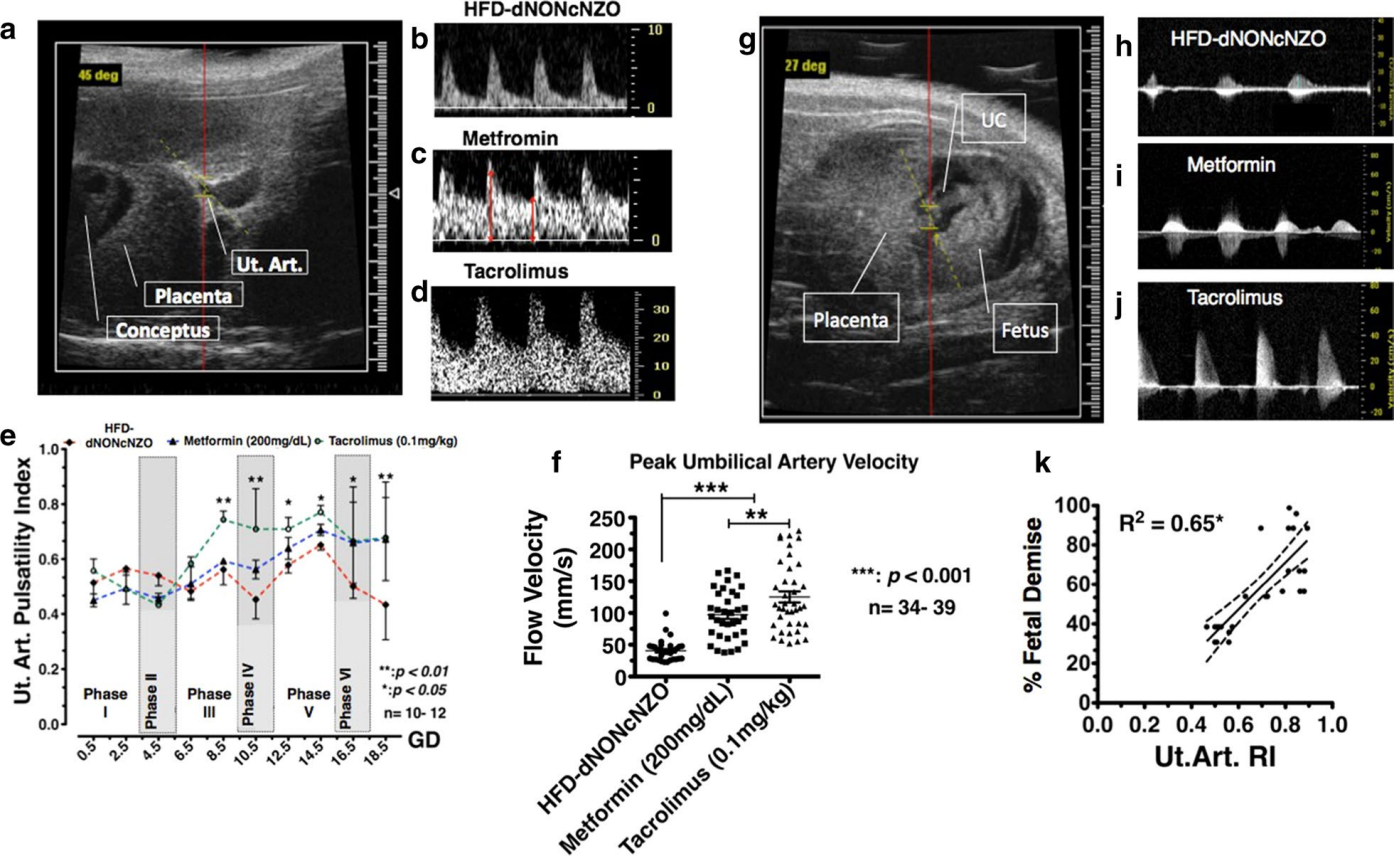
Tacrolimus-mediated inhibition of fetal demise is linked to restored uterine and umbilical artery flow during pregnancy. **a**–**d** Representative UBM image of the right uterine artery proximal to the conceptus being assessed at gd10.5 (**a**) and that of an Ut. Art. PWD in an HFD-dNONcNZO (**b**), tacrolimus-treated (**c**) or metformin-treated (**d**) (*long red arrow* peak systolic velocity, *short red arrow* end diastolic velocity). A significant reduction in the uterine artery blood flow was detected in the HFD-dNONcNZO dams between gd10.5-gd18.5 (**e**). **f** Group scatter-graphs of the peak umbilical arterial flow depicting impact of HFD and effect of treatment with metformin or tacrolimus in rescuing umbilical arterial flow in treated dams. **g** A representative UBM image taken from gd14.5 fetus and representative PWD for the most common differences in the umbilical artery peak flow velocity and waveform from HFD-dNONcNZO (**h**), metformin (**i**) or tacrolimus-treated dams (**j**). **k** A significant association between  % fetal demise and increased Ut. Art. resistivity during mid-late gestation was demonstrable in the HFD-dNONcNZO dams (**k**) (*p < 0.01). Note the significant increase in the umbilical arterial blood flow in the tacrolimus-treated dams over those receiving metformin (200 mg/dL) (**p < 0.01) (**e**). n = 7 dams/group and two experimental repeats with 34–39 pups examined in utero, *p* < 0.001 at 95% confidence

Maternal adaptation to pregnancy in the tacrolimus-treated HFD-dNONcNZO dams was further assessed to examine aspects of uterine (Ut. Art.) and umbilical artery flow dynamics during gestation using Doppler ultrasonography during early and late-mid gestation. As shown in Fig. 3, representative ultrasound B-mode (UBM) images of the right uterine artery proximal to the conceptus assessed at GD10.5 in an HFD-dNONcNZO dam (Fig. 3a, b) reveals consistent pattern of uterine artery insufficiency in the untreated dams as indicated by the UBM waveform and low peak systolic and end-diastolic flow velocities compared to those receiving metformin (Fig. 3c) or tacrolimus (Fig. 3d). Indeed, rescued Ut. Art. flow in the tacrolimus- or metformin-treated dams resulted in restored vascular pulsatility throughout gestation and at specific phases of pregnancy in treated dams as shown in Fig. 3e (comparing all experimental groups). Noticeably, similar to findings previously described [19], Ut. Art. PI segregated into phases corresponding to fluctuations in the normoglycemic mouse pregnancy and associated embryonic/fetal and maternal events, with phases 1, 2 depicting normal peri-implantation events, phases 3, 4 were the outcome of the placentation process and phases 5, 6 reflective of progressive fetal growth-associated vascular events. Additionally, microultrasonographic examination of fetuses belonging to the HFD-dNONcNZO dams revealed features of in utero fetal distress. Among the latter were the significantly restricted Ut.Art. flow (40.51 ± 2.73 mm/s in the HFD-dNONcNZO dams vs 125.14 ± 8.68 mm/s in the tacrolimus-treated and 97.13 ± 6.43 mm/s in the metformin-treated dams, respectively) (Fig. 3f), and features of cardiac dysrhythmias such as bradycardia detected in fetuses of untreated dams (Fig. 3g, and compare Fig. 3h–j, respectively). Interestingly, percentage of fetal demise was positively correlated with increased Ut. Art. resistivity during mid-late gestation in the untreated HFD-dNONcNZO dams (Pearson Correlation Coefficient = 0.65, with *p* < 0.01 when compared to the treated) (Fig. 3k).

#### Protection against fetal demise in the tacrolimus- and metformin-treated HFD-dNONcNZO dams is linked to proper spiral artery (SA) remodeling

**Fig. 4:**
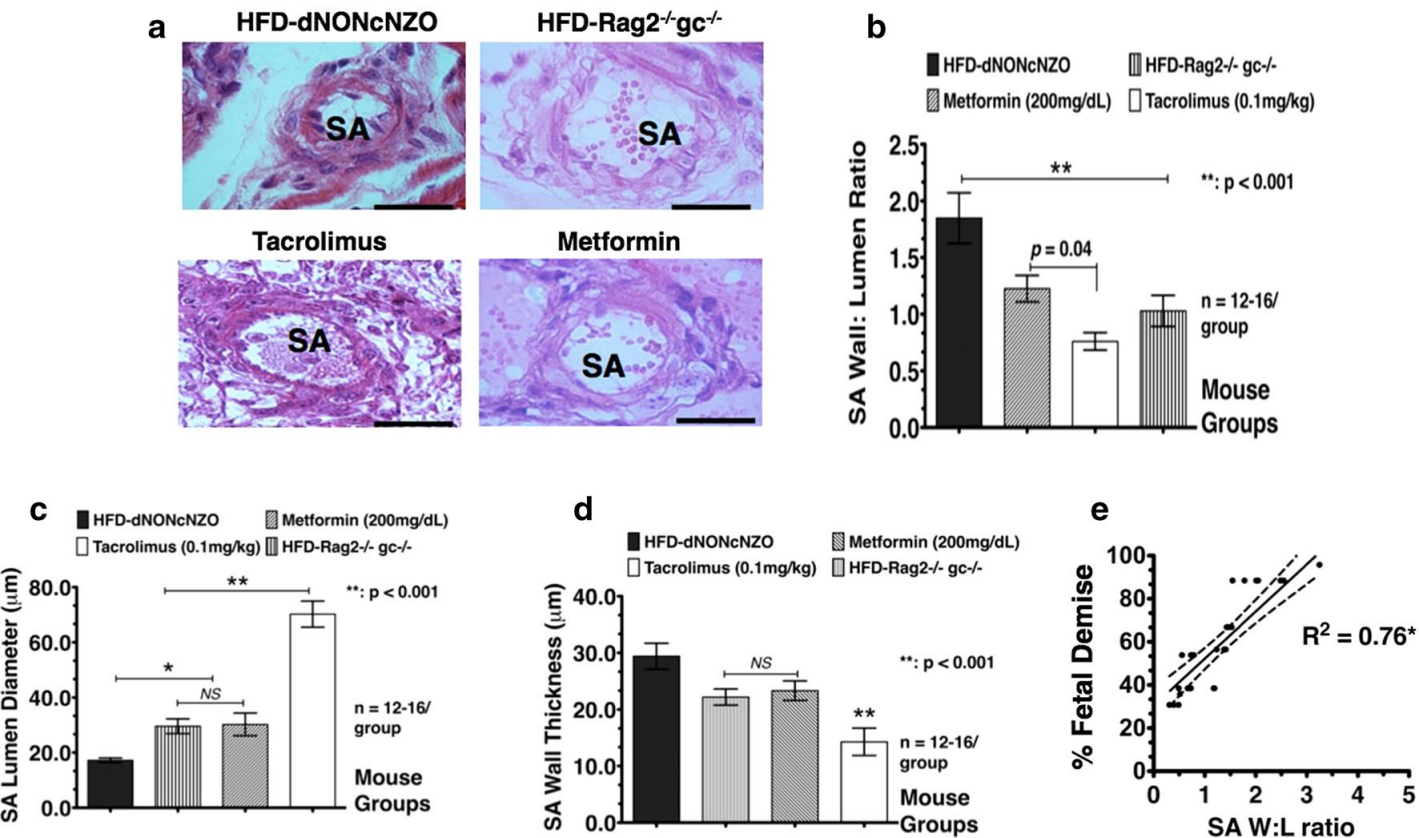
Low percentage of fetal demise in the tacrolimus-treated HFD-dNONcNZO dams is linked in part to restored spiral artery (SA) remodeling during gestation. **a** Representative spiral artery histopathological analysis at gd10.5 revealing restricted remodeling of these structures in the HFD-dNONcNZO dams as measured in their significantly higher wall:lumen ratio (**b**), narrower lumen (**c**) and thicker SA walls (**d**). Direct causal relationship existed between  % FGR and restricted SA W:L ratio at gd10.5 in the HFD-dNONcNZO dams (**e**). N = a minimum of 7 dams/group and two experimental repeats, *p < 0.01 at 95% confidence

Histopathological analysis of SA remodeling at GD10.5 revealed restricted process in the HFD-dNONcNZO dams as measured in their significantly higher wall: lumen ratio (Fig. 4a, b), narrower lumen (Fig. 4c) and thicker SA walls (Fig. 4d) when compared to treated dams or their normative controls. A significant direct casual association between % fetal demise as a selected APO and improper SA remodeling (Pearson Correlation Coefficient = 0.76 with *p* < 0.01 compared to untreated) was detected in the untreated HFD-dNONcNZO dams (Fig. 4e) suggesting a link between poor uterine arterial physiology and progeny outcomes in these mice.

### Tacrolimus and fetal parameters in treated HFD-dNONcNZO mice

#### External gross and skeletal malformations

**Fig. 5:**
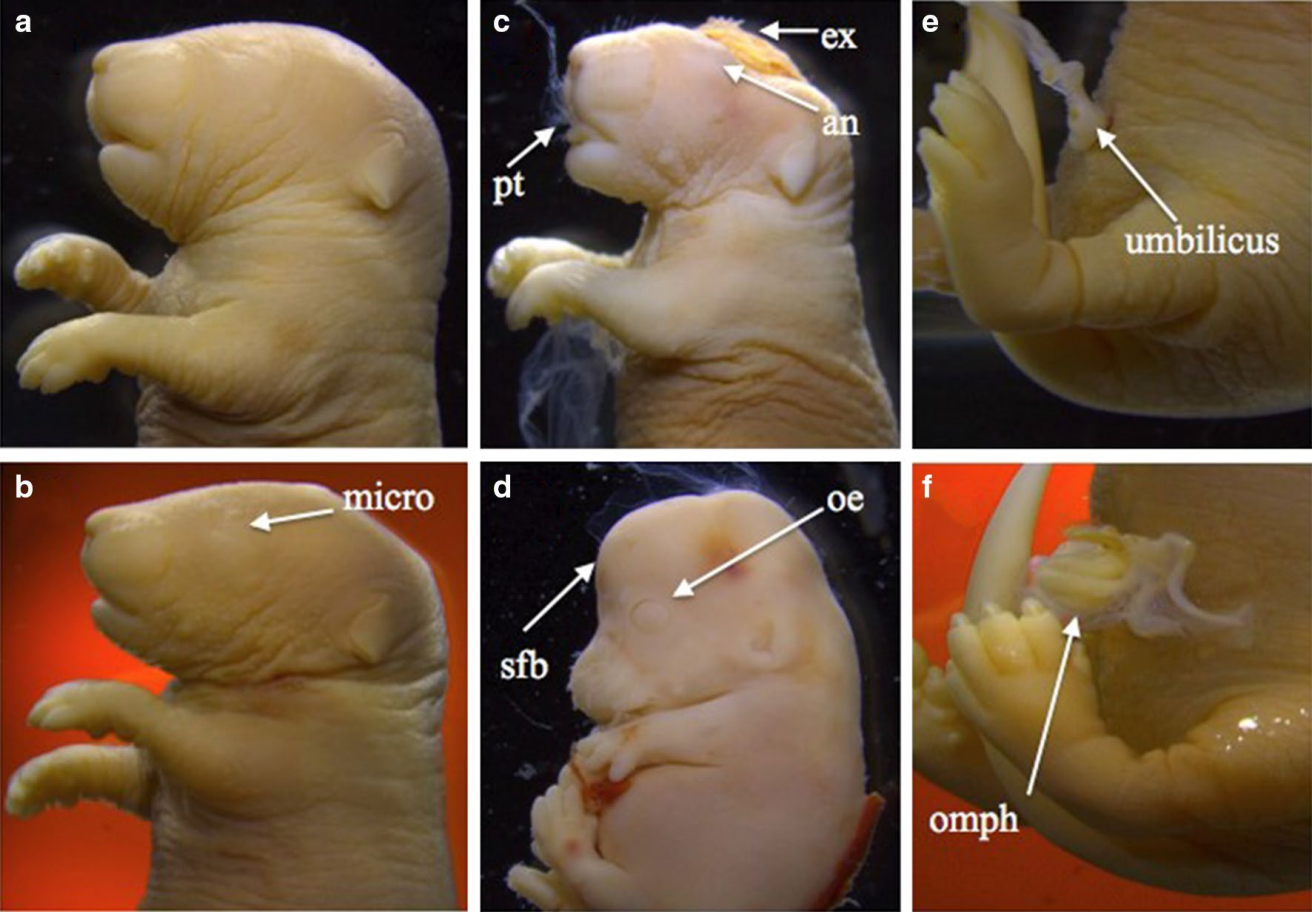
External features characteristics of HFD-dNONcNZO fetuses at the time of Caesarean section at GD 18.5. Although normal craniofacial and midline phenotypes were seen in less than 10% of the cases such as those in **a** and **e** respectively, microphthalmia (micro) in **b**, protruding tongue (pt), anophthalmia (an) and exencephaly (ex) in **c**, swollen forebrain (sfb) and open eye (oe) in **d**, and omphalocele (omph) in **f** were the most frequent defects in the offspring of diabetic HFD-dNONcNZO dams

Since high-fat diet (45% kCal fat) alters fetal growth and inhibits murine fetal bone development [27], we examined external and skeletal growth characteristics of fetuses delivered to HFD-dNONcNZO mice and those receiving metformin or tacrolimus. Due to the extremely low viability of offspring from HFD-dNONcNZO mice (*n* = 3), the external morphology was assessed in all fetuses (*n* = 10). The cumulative external malformation rate in the HFD-dNONcNZO offspring was 90% (Additional file 1: Table S2). Offspring from tacrolimus (0.05 or 0.1 mg/kg) and metformin treated mice had malformation rates of 5, 5.3 and 12%, respectively. Craniofacial defects were most prevalent and included in descending order of prevalence: anophthalmia, exencephaly, and protruding tongue. The midline defect, omphalocele was also noted in the HFD-dNONcNZO fetuses (Fig. 5).

**Fig. 6:**
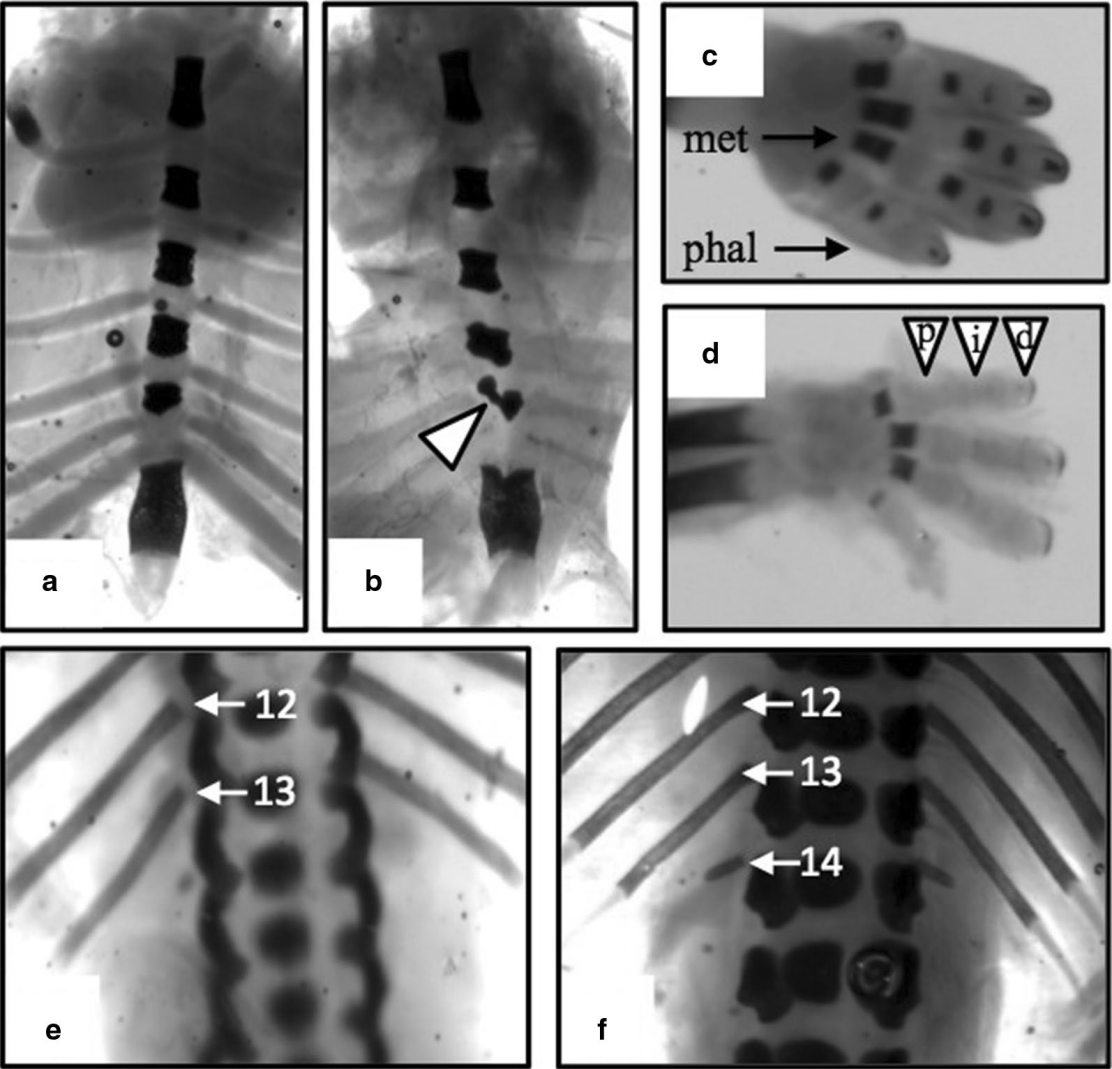
Representative depictions of common skeletal malformations and variations. Photomicrographs **a**, **b** illustrate, respectively, normal and misaligned ossification centers of the sternum (*arrowhead*). **c** Depicts ossification of the metacarpals (met) and phalanges (phal) in the forepaw, with delayed ossification of the proximal (), intermediate () and distal () phalanges shown in **d**, respectively. Ribs 12 and 13 are identified in **e** (*arrows*) with an additional 14th rib identified in **f** (*arrows*)

In assessing skeletal malformations only three fetuses were viable in all the HFD-dNONcNZO mice examined. One litter suffered a processing error and thus was not assessed. When exposed to tacrolimus or metformin, HFD-dNONcNZO mice displayed skeletal malformations, variations and ossification delays, but no pattern emerged (Table 2). The common malformation was misaligned sterna with an increased incidence in the number of fetuses with supernumerary ribs (a variation not a malformation; 14 instead of 13 ribs) and delayed ossification primarily in the distal limbs (Fig. 6).

**Table 2:** Summary of the major skeletal malformations, variations and delayed ossifications in fetuses examined

	HFD-dNONcNZO	Tacrolimus (0.05 mg/kg)	Tacrolimus (0.1 mg/kg)	Metformin (200 mg/dL)
Fetuses (litters)	3 (3)	22 (3)	18 (3)	17 (3)
14 ribs	33% (1)	27% (3)	44% (3)	18% (2)
Incomplete ossification				
Sternebra	^a^	0%	0%	6% (1)
Metacarpals	^a^	0%	0%	6% (1)
Forelimb phalanges	33% (1)	0%	6% (1)	12% (2)

Skeletons of viable fetuses were double-stained with Alcian blue and Alizarin red and assessed for malformations, variations and ossification delays. The incidences are reported in percentages with the values in parentheses reflecting the number of litters affected

^a^Litters were all resorbed or dead. Therefore no visceral or skeletal examinations were done on them (only external examination because the numbers of pups was so low)

#### Internal malformations

Internal examinations were only conducted on fetuses that were viable at the time of Cesarean section. Thus, only three HFD-dNONcNZO fetuses were examined, but each presented with multiple anomalies while the incidences of malformations in low-, high dose tacrolimus and metformin treated were 13.6, 11 and 5.9%, respectively (Additional file 1: Table S3). Anomalies included hydroureter, hydronephrosis, hydrocephalus, outflow tract anomalies and interventricular septal defect. Variations such as absent gallbladder, and deficient lobe number in right lung were also noted in the HFD-dNONcNZO offspring, but not the other groups (Additional file 1: Table S3).

## Discussion

The present study demonstrates that tacrolimus, an immunosuppressant currently used in organ transplant procedures, significantly improved glycemic control with concomitant improvement of reproductive outcomes, and increased postnatal survival without increasing risks of major congenital malformations in a murine model of human obesity-associated T2DM. In insulin resistant diabetic HFD-dNONcNZO mice, metformin normalized blood glucose levels to the same extent as tacrolimus but did not offer the same degree of protection against reproductive health and developmental adversities. This suggests that the immune system, independent of glycemic control, is a critical component of the management of adverse pregnancy outcomes associated with chronic maternal overnutrition.

Carrying a pregnancy after solid organ transplantation is considered a high risk for maternal, fetal and neonatal complications but has been successful. Multiple studies have demonstrated that clinically tacrolimus poses no increased risk of congenital malformations but may increase the incidence of low birth weight and pre-term delivery [10, 11, 28, 29]. These clinical experiences were in conflict, to some extent, with the preclinical safety studies conducted in female rats dosed prior to mating or during organogenesis [30, 31]. At a dose one-third of the maternally toxic dose based upon exposure, tacrolimus had adverse effects on female reproductive parameters causing embryolethality and significant reductions of pup weights [30]. These observations resulted in a US FDA pregnancy class C which states that “*Animal reproduction studies have shown an adverse effect on the fetus and there are no adequate and well*-*controlled studies in humans, but potential benefits may warrant use of the drug in pregnant women despite potential risks”.* However, in over 200 exposed pregnancies and 236 babies born to organ transplant recipients where tacrolimus was administered as a multi-drug based therapy, no significant teratogenicity could be concluded from multiple clinical registries [32–35]. Nonetheless, unlike the present study, preclinical safety reports in rats were in contradiction to each other. Saegusa and associates [30] reported on reduced reproductive performance, increased embryolethality and decreased pup weights among pregnant rats orally receiving 1.0 and 3.2 mg/kg FK506 (tacrolimus) after organogenesis and during lactation [30]. On the other hand, Farley and co-workers [31] recounted normal maternal health but dose-dependant elevation in the percentages of post-implantation resorption that led to the conclusion of a relative safety to the use of tacrolimus in pregnancy [31]. Similarly, Ramos et al. [36] reported on normal maternal and fetal health in otherwise normoglycemic female Wistar rats orally receiving tacrolimus at 10–40 times the currently described dosage. Notwithstanding species and route of administration related-differences in interpreting tacrolimus pharmacokinetics among mice, rats and women, our data are generally supportive of previous reports from clinical registry leveraging records from 20 to 200 women receiving 0.1–0.4 mg/kg/day tacrolimus mono-therapy or in a combination of immunosuppression whereby higher fetal exposure ratios were found to be exponentially related to higher maternal trough blood concentrations of the compound but reported on normal birth weights and post-natal development in these pregnancies [28, 35, 37–39]. This is an important consideration because tacrolimus, like most drugs, crosses the placenta with concentrations found in the cord blood being ~71, 23 and 19% of maternal concentrations for whole blood, plasma and unbound form, respectively [28, 39]. Even with transfer across the placenta, tacrolimus neither causes gross congenital malformations in humans [10, 32, 40, 41] nor does it in normoglycemic Wistar rats exposed for 15 days to about 10–40× the current dose *per oral* during peri-implantation phase [36]. Nevertheless, tacrolimus has been reported to cause transient and reversible materno-fetal comorbidities such as hyperkalemia and in some cases reduced renal mass (oligonephronia) thereby increasing the risk for further development of renal failure and hypertension in adult life [40, 42]. Additionally, it should be pointed out that although the present study used tacrolimus to normalize glycemic control in the obese and T2DM mice, a paradoxical new-onset diabetes after transplantation (NODAT), a severe complication following organ transplantation, has been reported to occur in between 2 and 53% of transplanted patients receiving higher dosing of tacrolimus and/or having pretransplantation hyperglycemia (reviewed in [43–45]. Nonetheless, NODAT has not been reported in recent studies on allogeneic uterine transplantation in rats receiving low dosing of tacrolimus [46], neither was it associated with worsening of clinical outcomes during a mean follow-up of 3 years in kidney transplant recipients [47]; nevertheless it is warranted that further pre-clinical safety studies on the potential use of tacrolimus as anti-diabetic agent should be conducted.

Among endometrial changes and mechanisms linked to the adverse pregnancy outcomes in diabetic mice and women are impaired spiral artery remodeling and trophoblast invasion defects during gestation [48, 49]. In an attempt to elucidate how tacrolimus restored vascular adaptation to pregnancy in the obese and diabetic mice, we examined uterine arterial physiology, spiral artery remodeling and late gestational (GD 18.5) placental cytokines during pregnancy in the HFD-dNONcNZO dams. Incomplete spiral artery remodeling with lumen stenosis has been recognized among ominous pathophysiological signs of poor placentation in GDM [50, 51], This heralding histopathological sign was evident in the placentas of HFD-dNONcNZO dams expressing higher levels of pro-inflammatory cytokines that include TNFα, IL16 and IL23 (Additional file 1: Table S4). This is consistent with the reported human and non-human primate data that the placenta develops exaggerated pro-inflammatory response to obesity, which contributes to or results from placental vascular insufficiency [52, 53]. Therefore, important implications came from findings of restricted uterine artery pulsatility and poor umbilical flow dynamics during gestation in the HFD-dNONcNZO dams. Vascular indices (RI and/or PI) are used to investigate impedance of the vascular bed distal to the vessel being examined, and a large quantity of continuous forward flow is generally observed throughout the diastole in low-resistance arterial waveforms [54]. Contrary to this, higher vascular indices are characteristics of vascular flow supplying high-resistance and leaky vascular beds [54]. Through mechanisms linked to chronic systemic hyperglycemia, local inflammation and the release of pro-inflammatory and pro-angiogenic molecules such as vascular endothelial growth factor (VEGF), advanced glycation end-products (AGE) and alterations to de novo synthesis of nitric oxide (NO), maternal hyperglycemia is known to induce vascular defects characterized by increased angiogenesis, increased vascular resistance, hyper-coagulability and preponderance of highly permeable vessels [55–59]. On the other hand, it has been shown that umbilical artery flow velocity negatively correlates with umbilical artery resistance and is reflective of alterations to the uterine artery PI and placental vascular resistance [60]. Therefore, the overall lower uterine artery PI throughout gestation and higher RI in the untreated HFD-dNONcNZO dams dictates their poor breeding performance and the higher rates of inflammation-mediated fetal demise observed in this mouse model. Also, considering that the placenta is an organ requiring constant perfusion and has low vascular indices with uterine artery PI values decreasing with gestational age [61], the present findings that therapeutic interventions with either tacrolimus or metformin normalized uterine artery PI in the treated HFD-dNONcNZO dams have clinical inferences suggestive of a pivotal role for adequate maternal glycemic control and immuno-modulation relative to pregnancy outcomes in conditions of chronic maternal overnutrition.

Taken together, the present study support the observation that adverse fetal health among neonates born to diabetic mothers cannot be explained solely on the basis of maternal hyperglycemic control [62]. Our data are also in parallel with the notions made by Leach et al. and Ericsson et al. that hyperglycemia should only be seen as a player within a spectrum of diabetic complications whereby maternal inflammation might have the upper hand [62, 63]. Nonetheless, the present data are in conflict with the primate studies reported by Frias and associates [52] where in the relatively wider placenta exchange area and high functional reserve are to be blamed for the lower rates of fetal growth restriction reported in the primate studies. However, the high rate of fetal demise at necropsy reported in the present study and by Frias and associates points to the uteroplacental flow restriction and inflammation to be the plausible culprits. Indeed, similar to the non-human primate model of obesity-induced placental growth restriction [52], our findings suggest that inflammation in the pregnant adult HFD-dNONcNZO female mice is likely localized in tissues involving at least the placenta. This supports previous studies in that the end-organ response to HFD is local yet associated with dysregulation of metabolic, vascular and inflammatory pathways requiring an immunosuppression [64].

A limitation of the present study is the chance of investigating renal functions among the tacrolimus-treated HFD-dNONcNZO dams. It has been reported in ordinary kibble-fed male Wistar rats that tacrolimus at a dose of 0.6 mg/kg/day for 9 consecutive weeks is nephrotoxic [65]. Important to tacrolimus pharmacokinetics and mechanism of action is its bioavailability and the ratio of bound:free form in the blood. The latter two maternal variables are greatly influenced by a multitude of factors among which the renal functions have long been recognized [66]. Therefore, future studies are warranted to establish whether or not our treated mice suffered tacrolimus-related nephrotoxicity. A possibility to overcome this limitation is the titration of dosage to maintain whole blood tacrolimus trough concentrations in the usual therapeutic range in plasma or urine of treated dams. This is particularly important since tacrolimus pharmacokinetic changes during pregnancy with maternal factors such as anemia and hypoalbuminemia significantly contribute to at risk pregnancy.

## Conclusions

Our data suggest a casual association between chronic maternal overnutrition and the development of materno-fetal comorbidities manifested in the immune-mediated glucose intolerance, defective spiral artery modification, placental insufficiency and adverse fetal outcomes in a mouse model of human obesity-associated T2DM. We have specifically found that the use of tacrolimus at the dose of 0.1 mg/kg q2d is adequate in restoring glucose homeostasis prior to and during gestation in the obese and diabetic subjects. The weight of evidence from pregnancies following solid organ transplant points to a marginal embryo-fetal safety of in utero tacrolimus exposure. On the other hand, metformin normalized glucose intolerance in HFD-dNONcNZO mice but offered less protection against diabetes-induced embryopathies than tacrolimus. This suggests that the etiology, incidence and severity of diabetes-associated congenital anomalies may have an immunological component requiring appropriate immuno-therapeutic intervention.

## Additional file

**Additional file 1.** Additional materials and methods, tables and figures.

## Abbreviations

T2DM: type 2 diabetes mellitus
APO: adverse pregnancy outcomes
HFD: high-fat diet
GDM: gestational diabetes mellitus
OGTT: oral glucose tolerance test
PWD: pulse wave Doppler
AUC: area under the curve
IFNγ: interferon gamma
TNFα: tumour necrosis factor-alpha
IL: interleukin
