# Supplementary material for: Tacrolimus in the prevention of adverse pregnancy outcomes and diabetes-associated embryopathies in obese and diabetic mice
Source: J Transl Med. 2017 Feb 13;15:32. doi: 10.1186/s12967-017-1137-4 (PMC5307666; doi:10.1186/s12967-017-1137-4)

Additional Materials and Methods, Tables and Figures

Tacrolimus in the Prevention of Adverse Pregnancy Outcomes and Diabetes-Associated Embryopathies in Obese and Diabetic Mice

By

Ahmad J. H. Albaghdadi^a^, Melanie A. Hewitt^a^, Samantha Putos^a^, Michael Wells^b^,Terence R. S. Ozolinš^a^, Frederick W. K. Kan^a,1^

^a^ Department of Biomedical and Molecular Sciences, Faculty of Health Sciences, Queen’s University, Kingston, Ontario, Canada K7L3N6

^b^ PARTEQ Innovations, Queen’s University, Kingston, Ontario, Canada K7L 0E9

^1^Corresponding author: Dr. Frederick W. K. Kan

Address: Department of Biomedical and Molecular Sciences, Faculty of Health Sciences, Queen’s University, Kingston, Ontario, Canada K7L3N6

Email: kanfwk@queensu.ca

Telephone: +1 613 533-2863

**Cytokine Proteome Profiler Array Analysis**

Quantification of placenta plasma levels of cytokines in the HFD-dNONcNZO dams and those receiving metformin (200mg/dL) or tacrolimus (0.1mg/Kg) relative to those of the HFD-Rag2^-/-^gc^-/-^ mice was performed using the Mouse Cytokine Proteome Profiler Array Panel A kit (R&D Systems) according to the manufacturer’s exact specifications. For maximum sensitivity, supernatants (500μl each) of samples were incubated with the supplied Cytokine Array Panel A Antibody Cocktail for 24 hour at 4°C under constant agitation. After blocking the array membranes with the supplied blocking buffers, supernatant-antibody mixtures were added to the membrane and blots were incubated overnight at 4°C on a shaking platform. Afterwards, membranes were washed in wash buffers before they were incubated with streptavidin-HRP [1:500 (vol/vol) in 5% (vol/vol) non-fat milk (Thermo Fisher, Canada) in PBBS-T] for 30 minutes at 23°C. Blots were then washed and Enhanced chemiluminescence incubation was conducted for 5 min using the SuperSignal West Femto chemiluminescent kit (Thermo Scientific, Canada). Images were then captured and analyzed using Alpha-Innotech H2D Imager coupled with AlphaEaseFC software (version 4.1, Alpha Innotech, San Leandro, CA). Fold change in placental cytokine expression was calculated using the equation “Fc = log2 (Eka)- log2 (Ekb)” where ‘Eka’ is the expression level of the control dams and Ekb is that of the test group.

**Additional Tables Legends**

**Additional file 1: Table S1. Mouse models, therapeutic interventions, dietary and husbandry conditions reported in this study**

| **Mouse Model** | **Source** | **Diet** | **Therapeutic Intervention** | **Numbers** |
| --- | --- | --- | --- | --- |
| **NONcNZO10/LtJ** | The Jackson Lab. (stock # 004456M) | 60% kCal high fat diet (D12492, Research Diets Inc., NJ, USA) | Vehicle  (castor oil: ethanol mix) | 14 |
| **NONcNZO10/LtJ** |  |  | Tacrolimus (0.1mg/kg s.c. q2d)* | 14 |
| **NONcNZO10/LtJ** |  |  | Tacrolimus (0.05mg/kg s.c. q2d)* | 14 |
| **NONcNZO10/LtJ^a^** |  |  | Metformin (200mg/dL) | 14 |
| **BALB/c-Rag2^-/-^Il2rg^-/-b^** | Queen’s University Animal Care Services |  | Vehicle | 12 |
| **BALB/c-Rag2^-/-^Il2rg^-/-b^** |  | 20% fortified protein pellet diet (5K52 LabDiet^®^) | Vehicle | 12 |

- * Administered on an alternate day regimen from week 15-18 and week 25-28, respectively.
- **^a^** Those receiving metformin referred to as drug-treated control
- **^b^** Normoglycemic (normative) control
- Mice were housed in a decontaminated barrier facility under standard sanitation, disinfection and sterilization practices as approved by Queen’s University Animal Care Services.

The animal room environment and photoperiod were as follows: temperature 20 ± 3**°**C; humidity 30% to 70%; 12hour light/dark photoperiod (lights on at 07:00AM), except when room lights were turned on during the dark cycle to accommodate blood sampling.

**Additional file 1: Table S2. External gross malformation in HFD-NONcNZO mice and those receiving tacrolimus or metformin**


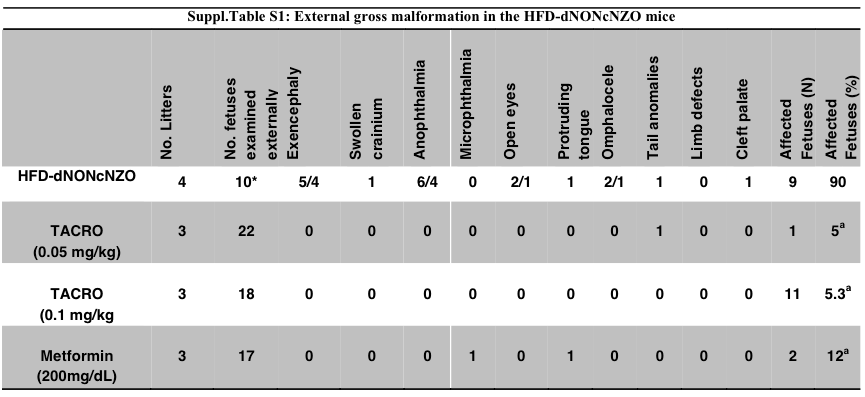


*: Only 3 fetuses were viable in the untreated group therefore, all non-resorbed foetuses were assessed for external malformations, whereas external malformations were only reported for the viable fetuses in the other treatments.

Parentheses indicate the number of litters affected.

^a^ statistically significant different from respective diabetic strain p<0.05 Kruskal-Wallis

**Additional file 1: Table S3. Visceral findings in the HFD-dNONcNZO mice and those receiving tacrolimus or metformin**

|  | | | | | | | | | | | | | |
| --- | --- | --- | --- | --- | --- | --- | --- | --- | --- | --- | --- | --- | --- |
|  | **No. Litters** | **No. fetuses examined** | **Gall bladder absent** | **Less than 3 liver lobes on R side** | **Hydroureter** | **Hydronephrosis** | **Hypotrophic kidney** | **Less than 4 lung lobes R side** | **Outflow tract anomaly** | **Interventricular septal defect** | **Hydrocephalus** | **No. of Embryos with visceral malformation** |  |
| HFD-dNONcNZO | **3** | **3*** | **(33%) 1** | **(33%) 1** | **(33%) 1** | **(33%) 1** | **0** | **0** | **(33%) 1** | **(33%) 1** | **(2.6%) 1** | **(100%)^a^ 3** |  |
|  | **3** | **22** | **0** | **0** | **0** | **(4.5%) 1** | **(4.5%) 1** | **0** | **(4.5%) 1** | **0** | **0** | **(13.6%)^a^ 3** |  |
| TACRO (0.05 mg/kg) |  |  |  |  |  |  |  |  |  |  |  |  |  |
|  |  |  |  |  |  |  |  |  |  |  |  |  |  |
|  | **3** | **18** | **0** | **0** | **(5.6%) 1** | **(5.6%) 1** | **0** | **0** | **0** | **(5.6%) 1** | **0** | **(11%)^a^ 2** |  |
| TACRO (0.1 mg/kg) |  |  |  |  |  |  |  |  |  |  |  |  |  |
|  |  |  |  |  |  |  |  |  |  |  |  |  |  |
|  | **3** | **17** | **0** | **0** | **(5.9%) 1** | **(5.9%) 1** | **0** | **0** | **0** | **0** | **0** | **(5.9%)^a^ 1** |  |
| Metformin (200mg/dL) |  |  |  |  |  |  |  |  |  |  |  |  |  |
|  |  |  |  |  |  |  |  |  |  |  |  |  |  |

(N)N = (percent of viable embryos) number of litters affected

^a^ statistically different from respective viable diabetic strain *p*<0.05 Kruskal-Wallis

*: most pups were resorbed at necropsy so that no visceral examination could be conducted on them.

**Additional file 1: Table S4. Placental plasma levels of cytokines in the HFD-dNONcNZO dams receiving vehicle compared to tacrolimus (0.1mg/kg sc q2d) or metformin (200mg/dL/day) at GD14.5**

|  |  |  |  | ***P* value** | |
| --- | --- | --- | --- | --- | --- |
| **Target** | **HFD** | **TACRO** | **METFORMIN** | **TACRO**  **vs HFD** | **Metformin**  **vs HFD** |
| **MCP1 (JE) (CCL2)** | 1.07 ± 0.26 | 0.59 ± 0.12 | 0.47 ± 0.14 | 0.26 | 0.34 |
| **MIP-1a (CCL3)** | 1.75 ± 0.43 | 2.48 ± 0.62 | 2.78 ± 0.69 | 0.52 | 0.36 |
| **MIP-1b (CCL4)** | 2.94 ± 0.73 | 5.06 ± 1.26 | 2.11 ± 0.52 | 0.06 | 0.29 |
| **MIP-2** | 0.28 ± 0.11 | 0.49 ± 0.13 | 0.24 ± 0.17 | 0.08 | 0.47 |
| **RANTES (CCL5)** | 0.18 ± 0.08 | 3.32 ± 0.11 | 0.17 ± 0.08 | 0.015* | 0.41 |
| **Eotaxin (CCL11)** | 2.01 ± 0.51 | 2.69 ± 0.67 | 1.56 ± 0.39 | 0.18 | 0.26 |
| **MCP5 (CCL12)** | 0.42 ± 0.13 | 0.55 ± 0.08 | 0.33 ± 0.09 | 0.25 | 0.31 |
| **TARC (CCL17)** | 0.78 ± 0.17 | 4.32 ± 0.14 | 0.54 ± 0.06 | 0.012* | 0.20 |
| **MIG (CXCL9)** | 1.45 ± 0.36 | 0.76 ± 0.19 | 0.69 ± 0.17 | 0.21 | 0.34 |
| **ITAC (CXCL11)** | 1.02 ± 0.27 | 1.97 ± 0.49 | 1.01 ± 0.25 | 0.09 | 0.28 |
| **SDF-1 (CXCL12)** | 2.10 ± 0.52 | 1.28 ± 0.32 | 0.82 ± 0.21 | 0.37 | 0.35 |
| **IP10** | 2.42 ± 0.61 | 1.47 ± 0.36 | 0.94 ± 0.24 | 0.36 | 0.34 |
| **KC** | 1.04 ± 0.26 | 0.64 ± 0.16 | 0.39 ± 0.19 | 0.36 | 0.41 |
| **IFNγ** | 1.18 ± 0.29 | 1.17 ± 0.29 | 0.78 ± 0.22 | 0.95 | 0.35 |
| **TNF-a** | 7.66 ± 0.66 | 0.59 ± 0.15 | 0.63 ± 0.37 | 0.002* | 0.003* |
| **IL-16** | 5.11 ± 0.77 | 0.75 ± 0.43 | 1.22 ± 0.31 | 0.017* | 0.07 |
| **IL1a** | 2.06 ± 0.51 | 3.33 ± 0.83 | 2.85 ± 0.71 | 0.27 | 0.32 |
| **IL1b** | 1.66 ± 0.41 | 2.79 ± 0.69 | 1.13 ± 0.28 | 0.33 | 0.64 |
| **IL2** | 1.62 ± 0.40 | 2.47 ± 0.61 | 0.85 ± 0.21 | 0.49 | 0.05 |
| **IL-3** | 1.85 ± 0.46 | 2.71 ± 0.67 | 0.86 ± 0.22 | 0.56 | 0.51 |
| **IL-4** | 1.15 ± 0.28 | 2.39 ± 0.34 | 2.05 ± 0.51 | 0.41 | 0.32 |
| **IL-5** | 2.79 ± 0.69 | 4.03 ± 1.0 | 4.35 ± 1.08 | 0.05 | 0.05 |
| **IL-6** | 2.39 ± 0.59 | 4.25 ± 0.81 | 3.92 ± 0.98 | 0.36 | 0.25 |
| **IL-7** | 0.67 ± 0.16 | 3.07 ± 0.26 | 0.62 ± 0.15 | 0.12 | 0.23 |
| **IL-10** | 2.27 ± 0.57 | 3.09 ± 0.77 | 3.72 ± 0.93 | 0.36 | 0.25 |
| **IL-13** | 0.90 ± 0.23 | 1.34 ± 0.33 | 0.79 ± 0.19 | 0.27 | 0.37 |
| **IL-1ra** | 2.13 ± 0.53 | 3.67 ± 0.92 | 2.74 ± 0.68 | 0.28 | 0.54 |
| **IL-12 p70** | 2.94 ± 0.74 | 3.44 ± 0.86 | 5.37 ± 1.34 | 0.27 | 0.07 |
| **IL-17** | 1.01 ± 0.25 | 1.75 ± 0.44 | 1.27 ± 0.32 | 0.28 | 0.31 |
| **IL-23** | 8.46 ± 0.61 | 3.72 ± 0.93 | 3.67 ± 0.91 | 0.037* | 0.041* |
| **IL-27** | 1.37 ± 0.34 | 1.83 ± 0.45 | 2.29 ± 0.57 | 0.47 | 0.34 |
| **BCL** | 1.27 ± 0.32 | 2.28 ± 0.57 | 1.54 ± 0.39 | 0.23 | 0.28 |
| **C5a** | 11.73 ± 0.43 | 1.17 ± 0.45 | 7.80 ± 0.29 | 0.027* | 0.19 |
| **G-CSF** | 1.28 ± 0.32 | 8.3 ± 0.58 | 1.27 ± 0.31 | 0.031* | 0.33 |
| **GM-CSF** | 2.45 ± 0.61 | 6.26 ± 1.36 | 7.09 ± 0.77 | 0.07 | 0.13 |
| **M-CSF** | 0.65 ± 0.16 | 0.43 ± 0.21 | 0.66 ± 0.16 | 0.36 | 0.38 |
| **TIMP-1** | 0.41 ± 0.09 | 0.24 ± 0.06 | 0.35 ± 0.08 | 0.21 | 0.30 |
| **sICAM1 (CD54)** | 0.56 ± 0.14 | 0.33 ± 0.08 | 0.43 ± 0.11 | 0.39 | 0.44 |

Placenta Cytokine Proteome Profiler^TM^ Array. Arbitrary units presented as mean ± S.E.M. * indicates a significant change as determined by Neuman-Keuls test; *p* < 0.05. n = 4/group.

**Additional Figures Legends**

**Additional file 1: Fig.S1.** Schematic depicting the focus of the study and the temporal sequence of experimental procedures including administration of high-fat diet, tacrolimus, and metformin as well as establishment of pregnancy. *Tacrolimus monotherapy was administered to diabetic HFD-dNONcNZO mice on an alternate day regimen for four consecutive weeks spanning the period of the HFD-weeks 15-18 and 25-28, respectively. OGTT: Oral glucose tolerance test. C/S: Cesarean section.

**
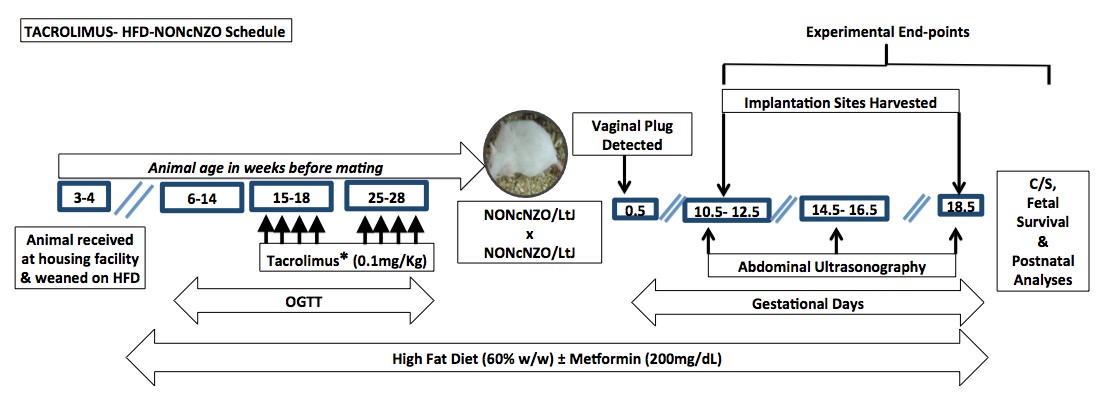
**

**Additional file 1: Fig.S2. A-C:** Temporal alterations to the Area Under the Curve (AUC) for glucose at OGTT obtained at 7 Wk HFD (**A**), 15 Wk HFD **(B)** or 25 Wk of HFD (**C**), respectively. Note the lack of changes to AUC glucose at OGTT in the alymphoid mice throughout the experimental timeline and the effect of two therapeutic interventions of tacrolimus (0.1mg/kg sc q2d) each of four consecutive weeks in restoring reference values of glucose despite HFD chronicity in treated dams (**A-C**). Asterisks indicate levels of significant differences at **p* ≤ 0.05, ***p* ≤ 0.01 and *** *p* ≤ 0.001. *ns*: not statistically significant.


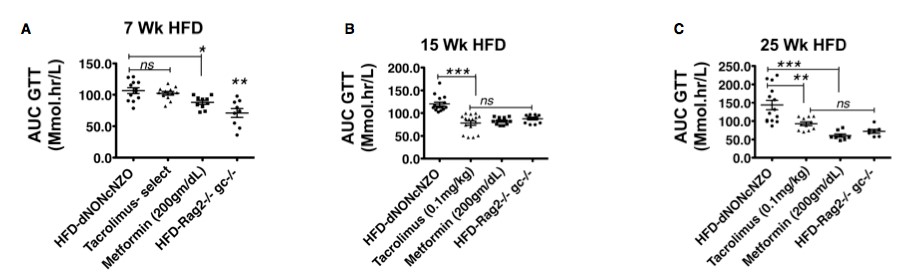

Supplement: Supplementary file 1 — Additional file 1. Additional materials and methods, tables and figures. [file 12967_2017_1137_MOESM1_ESM.docx]
